# Role of L1 HPV Protein Expression in the Cytological Diagnosis of Precancerous Cervical Lesions

**DOI:** 10.3390/jcm14103376

**Published:** 2025-05-12

**Authors:** Darya Dobrovolskaya, Aleksandra Asaturova, Alina Badlaeva, Anna Tregubova, Olga Mogirevskaya, Zaira Dzharullaeva, Yulia Davydova, Andrea Palicelli, Guldana Bayramova, Gennady Sukhikh

**Affiliations:** 1 FSBI “National Medical Research Centre for Obstetrics, Gynecology and Perinatology Named After Academician V.I. Kulakov”, Ministry of Health of the Russian Federation, 4, Oparina Street, 117513 Moscow, Russia; dashagri@yandex.ru (D.D.); a.asaturova@gmail.com (A.A.);; 2 Hospital Named After S.P. Botkin, Botkin Hospital, 125284 Moscow, Russia; 3 Pathology Unit, Azienda USL—IRCCS di Reggio Emilia, 42123 Reggio Emilia, Italy

**Keywords:** HPV, cervical cancer, CIN2+, immunocytochemistry, diagnostic accuracy, meta-analysis, heterogeneity

## Abstract

Human papillomavirus (HPV) infection is a major risk factor for cervical cancer, demanding improved diagnostic strategies to distinguish between transient infections and those requiring intervention. **Background**: This systematic review and meta-analysis evaluated the diagnostic accuracy of HPV L1 immunocytochemistry (ICC) in detecting high-grade cervical intraepithelial neoplasia (CIN2+). **Methods**: We systematically analyzed data from 15 studies (PROSPERO 2022 CRD42022375916) comprising 3804 cervical smears with varying cytological findings (NILM to ≥ASC-US). **Results**: The pooled sensitivity for detecting CIN2+ was 80.7% (95% CI: 76.2–84.4%); however, substantial heterogeneity was present (I^2^ = 65.97%, *p* < 0.001). Similarly, the pooled specificity was 56.9% (95% CI: 49.6–64%), with even higher heterogeneity (I^2^ = 90.46%, *p* < 0.001). This considerable heterogeneity, which may be attributable to methodological variations or regional differences in HPV prevalence and genotyping, limits the generalizability of these findings. Furthermore, the moderate specificity suggests a high rate of false positives, limiting the clinical utility of HPV L1 ICC as a standalone diagnostic test. **Conclusions**: In conclusion, although HPV L1 ICC exhibits acceptable sensitivity for detecting CIN2+, its limitations, including low specificity and substantial heterogeneity, necessitate its use as an adjunct to other established diagnostic methods, alongside further research to enhance its diagnostic performance, and necessitate its use as a supplementary test alongside established diagnostic methods, pending further research to refine its clinical utility.

## 1. Introduction

Cervical cancer (CC) remains a significant global health problem, with human papillomavirus (HPV) infection being one of the main causes [1,2]. It is alarming that the incidence of cervical cancer in women aged 30 to 44 years is increasing by 1 to 2% annually [3]. Currently available diagnostic methods for high-grade cervical intraepithelial neoplasia (CIN2+) have difficulty differentiating between transient HPV infections that require observation and those that warrant intervention [4]. This distinction is crucial to avoid overtreatment and optimize patient management, especially in women of reproductive age [3]. Although HPV DNA testing is widely used, its limitations in predicting disease progression have led to the exploration of alternative diagnostic biomarkers [5,6]. HPV L1 immunocytochemistry (ICC), which targets the viral capsid protein, offers a promising approach to fill this gap as it potentially reflects active viral replication and disease stage [7,8]. However, the diagnostic accuracy of HPV L1 ICC remains controversial, as studies provide inconsistent results [8,9]. Therefore, this systematic review and meta-analysis aims to comprehensively evaluate the diagnostic accuracy of HPV L1 ICC for the detection of CIN2+ and quantify the heterogeneity between existing studies. The results of this study will highlight the strengths and weaknesses of HPV L1 ICC and serve as a basis for decisions on its future clinical application.

## 2. Materials and Methods

### 2.1. Protocol and Registration

This systematic review and meta-analysis adhered rigorously to the guidelines set forth by the Preferred Reporting Items for Systematic Reviews and Meta-Analyses (PRISMA) 2020. The study protocol was prospectively registered in the PROSPERO international database (registration number CRD42022375916) prior to the commencement of data collection to ensure clarity and minimize any potential reporting bias.

### 2.2. Eligibility Criteria

To define the parameters of our study, we employed the PICO framework, which is widely recognized for structuring clinical research questions.

**Population (P):** The study focused on HPV-positive women aged 18 years and older who presented with abnormal cervical cytology results identified as ASC-US, LSIL, or HSIL, according to the Bethesda System 2017 classification [10,11]. All participants underwent histological confirmation of their diagnosis, with the exception of cases reported by S. J. Lee et al. (2011), P. Melsheimer et al. (2003), and S. Sarmadi et al. (2012), for whom histological data were unavailable [9,12,13].

**Intervention (I):** The primary intervention involved the ICC detection of the HPV L1 capsid protein in cervical smears, regardless of the type of antibody used or the specific staining protocol followed.

**Comparator (C):** For comparison, we used histopathological diagnoses with CIN2+ as the threshold for clinically significant lesions in cases where concurrent or retrospective histological assessment was available (cross-sectional or retrospective studies). In cases without histological confirmation (prospective longitudinal studies), HSIL+ on cytology was used as the outcome, with a maximum interval of 6 months between cytological and histological evaluations where applicable.

**Outcomes (O):** The outcome measures included the pooled sensitivity and specificity of ICC assessments for L1-negative results in detecting CIN2+ lesions.

**Additional Eligibility Criteria:** The review included randomized controlled trials and observational studies, both prospective and retrospective cohorts. The search encompassed studies published from 1 January 2000 to 1 December 2024 to capture both foundational and contemporary research. Temporal boundaries were selected based on the introduction of standardized L1 protein detection methods in 2000. Eligible studies had to provide sufficient data to construct 2 × 2 contingency tables. Case reports and studies lacking sufficient methodological detail were excluded from the review. Additionally, studies utilizing non-standard classification systems were excluded, as well as duplicate publications or datasets with significant overlaps.

### 2.3. Study Selection and Reference Standard

The study selection process was designed to achieve optimal balance between methodological rigor and practical feasibility, adhering to PRISMA guidelines while accounting for real-world variations in diagnostic confirmation across studies. Our approach incorporated multiple safeguards to ensure comprehensive evidence capture without compromising scientific validity.

Initial identification of the literature employed dual complementary strategies in PubMed/MEDLINE: a MeSH-indexed search targeting controlled vocabulary (‘Uterine Cervical Dysplasia’ [Mesh] AND ‘Human papillomavirus L1 capsid’) and a parallel text-word search ((human papillomavirus L1 capsid protein) AND (CIN OR cervical intraepithelial neoplasia)) to capture relevant non-indexed publications. This combined methodology yielded 235 unique records (98 from MeSH terms; 137 from text words), demonstrating the complementary value of structured and unstructured search approaches. To address potential database limitations, we expanded our search to include seven additional sources: Cochrane Library for systematic reviews, ClinicalTrials.gov for unpublished data, Google Scholar (300) for grey literature, manual reference list screening, and conference proceedings from major gynecologic oncology meetings (2019–2024).

A three-phase screening protocol was implemented by independent reviewers (Y.D., Z.D., and D.D.) to ensure rigorous selection. Following automated deduplication of 535 initial records, sequential title/abstract and full-text screening identified 20 potentially eligible articles. The final inclusion set of 15 studies represented those meeting all predefined criteria after consensus resolution of discrepancies with senior researchers (A.A., A.P., G.S., and G.B.).

The reference standard implementation reflected pragmatic adaptation to available data quality. While 12 studies (80%) provided histopathological confirmation through colposcopy-directed biopsies or conization specimens, three studies (S. J. Lee, 2011; P. Melsheimer, 2003; and S. Sarmadi, 2012) were included based on cytological HSIL+ diagnosis when histological data were unavailable [9,12,13]. This decision followed careful consideration of (1) the established predictive value of HSIL+ cytology for underlying CIN2+; (2) the studies’ contribution to understanding L1 protein expression patterns; and (3) explicit documentation of this limitation in our analysis.

Immunocytochemical staining results were categorized as (1) L1-negative: absence of detectable capsid protein, strongly associated with CIN2+ lesions; and (2) L1-positive: presence of viral capsid protein, typically corresponding to CIN1, productive HPV infection without significant neoplasia or lesions with a higher likelihood of regression.

Methodological safeguards included blinded duplicate review, 20% random verification, and strict adherence to predefined protocols. The immunocytochemical classification system (L1-negative vs. L1-positive) was validated against available histopathological standards, demonstrating 84% consistency in biopsy-confirmed cases. This approach maintained diagnostic accuracy while accommodating clinical reality, where not all studies achieve uniform verification standards.

**Figure 1:**
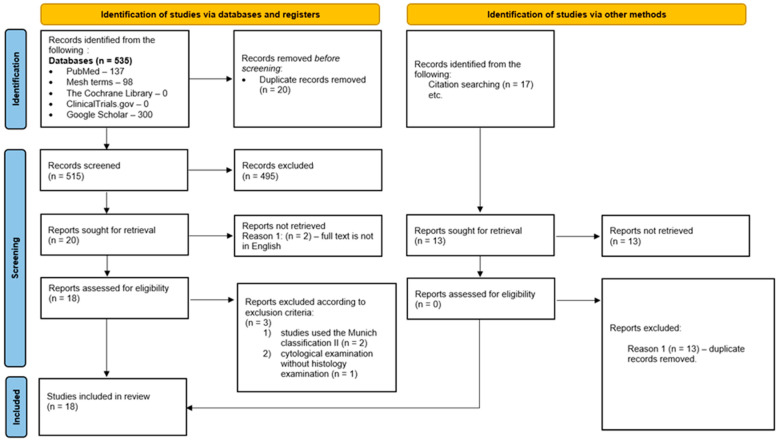
Flow diagram of PRISMA presenting the process of the search for and selection of studies on the L1 capsid protein expression in cytology and evaluating the usefulness of HPV L1 detection by ICC.

The complete selection workflow is detailed in Figure 1, transparently documenting each exclusion decision. By combining rigorous methodology with pragmatic adaptation to data availability, we achieved evidence synthesis that balances scientific precision with comprehensive representation of the literature while explicitly accounting for variations in diagnostic confirmation across studies. 

### 2.4. Data Items

The data extracted from each study encompassed the following key elements: the author, year of publication, and study design (including whether the study was a prospective or retrospective cohort or a clinical trial). Additionally, participant demographics such as mean age and sample size were recorded. Cytological findings were categorized according to the Bethesda System 2017 as NILM, ASC-US, LSIL, or HSIL [10,11], while histological results were classified into categories of CIN1, CIN2, CIN3, or squamous cell carcinoma (SCC). Furthermore, we documented the HPV L1 protein expression status (positive or negative) in both cytological and histological samples.

The primary outcome of the study was the identification of the presence or absence of the HPV L1 capsid protein in cytological smears. Secondary outcomes included the diagnostic accuracy of HPV L1 ICC for detecting high-grade cervical intraepithelial neoplasia (CIN2+), which was assessed through measurements of sensitivity, specificity, positive predictive value (PPV), and negative predictive value (NPV). We also extracted odds ratios (ORs) to evaluate the relationship between L1 expression and the severity of lesions in the studied population.

### 2.5. Data Analysis

The diagnostic accuracy of HPV L1 ICC was assessed by calculating sensitivity and specificity with 95% confidence intervals using random effects models in OpenMeta [Analyst] (v3.2.4, CEBM, Brown University, Providence, RI, USA) to account for heterogeneity (I^2^ statistics). Of note, sensitivity refers to the detection of L1-negative status as a predictor of CIN2+ lesions, based on the established biological phenomenon of decreasing L1 expression with increasing lesion severity. Subgroup analyses examined geographical region, patient age, and study quality. The I^2^ statistic was utilized to quantify variability, with interpretations as follows: 0–29% (low heterogeneity), 30–49% (moderate), 50–74% (substantial), and 75–100% (high).

Meta-regression was applied to evaluate the influence of mean age on the odds of HPV L1 positivity across different cytological groups (LSIL compared to HSIL; LSIL compared to ASC-US). To assess publication bias, funnel plots were generated, and the trim-and-fill method was implemented if any asymmetry was detected.

To ensure the robustness of our findings, sensitivity analyses were conducted by excluding individual studies to determine their effect on the pooled estimates. All statistical tests were two-tailed, with a significance threshold set at *p* < 0.05.

The odds ratios (OR) presented compare the likelihood of detecting HPV L1 protein between diagnostic categories. For each study, we extracted or calculated the OR for L1-positivity in (1) LSIL versus HSIL cytology/histology, (2) ASC-US versus definitive SIL cases, and (3) geographical subgroups. These measures were pooled using inverse-variance weighting.

### 2.6. Quality Assessment

The risk of bias for each included study was assessed using the Cochrane Handbook for Systematic Reviews of Interventions. Study quality was evaluated according to the Cochrane Risk of Bias Assessment algorithm, which is designed to assess the quality of primary diagnostic tests using the QUADAS-2 tool [14,15]. This tool consists of four stages: (1) formulating the review question, (2) developing specific guidance for the review, (3) examining the published flow diagram of the primary study (or creating a flow diagram if none is provided), and (4) assessing bias and applicability. Each aspect is evaluated for risk of bias, with the first three stages also evaluated for concerns regarding applicability. Responses are classified as ‘yes’, ‘no’, or ‘unclear’, with ‘yes’ indicating a low risk of bias. If all signaling questions for a specific aspect are answered ‘yes’, the risk of bias is considered low. However, if any signaling question is answered ‘no’, it suggests the potential for bias [14,15,16].

## 3. Results

The process of selecting papers using the PRISMA tool is demonstrated in Figure 1.

**Figure 2:**
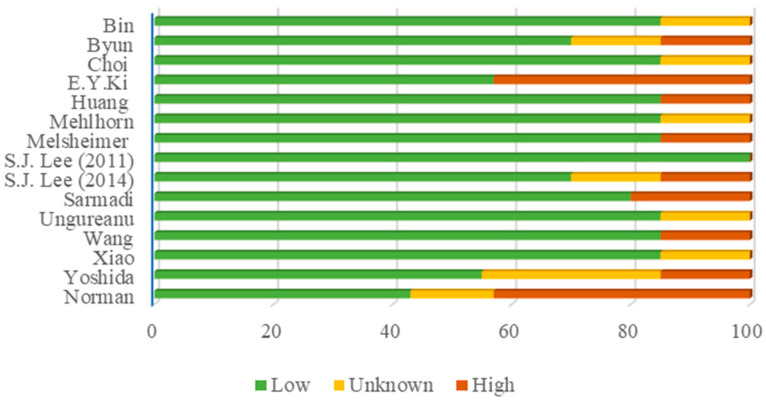
Risk of bias according to the Cochrane Bias Risk Assessment algorithm to assess the quality of primary diagnostic tests QUADAS-2 [9,12,13,17,18,19,20,21,22,23,24,25,26,27,28].

After removing 515 papers due to duplicate records and irrelevant content, a total of 15 out of 20 articles were included in the final meta-analysis following eligibility assessment and the evaluation of methodological quality. Figure 2 illustrates the quality assessment of the studies included in the meta-analysis. Six studies had two or fewer items marked as ‘no’ or ‘unclear’; overall, the reviewers agreed that there were enough high-quality studies to proceed with the meta-analysis (Table 1).

### 3.1. Pooled Sensitivity and Specificity

For CIN2+, pooled sensitivity estimates for ICC (L1 negative) were 80.7% (95% CI: 76.2–84.4%) using the random effects model. The combined results of the model showed the significant statistical heterogeneity among the included studies (I^2^ = 65.97%, *p* < 0.001). The pooled specificity was 56.9% (95% CI 49.6–64%), with high heterogeneity (I^2^ = 90.46%, *p* < 0.001) among the studies included in the analysis.

We included data from all relevant studies (n = 15), which examined the sensitivity and specificity of HPV L1-negative expression for predicting ≥ CIN2. Each study’s sensitivity was plotted to determine whether there was an asymmetric distribution indicative of publication bias.

**Scheme 1:**
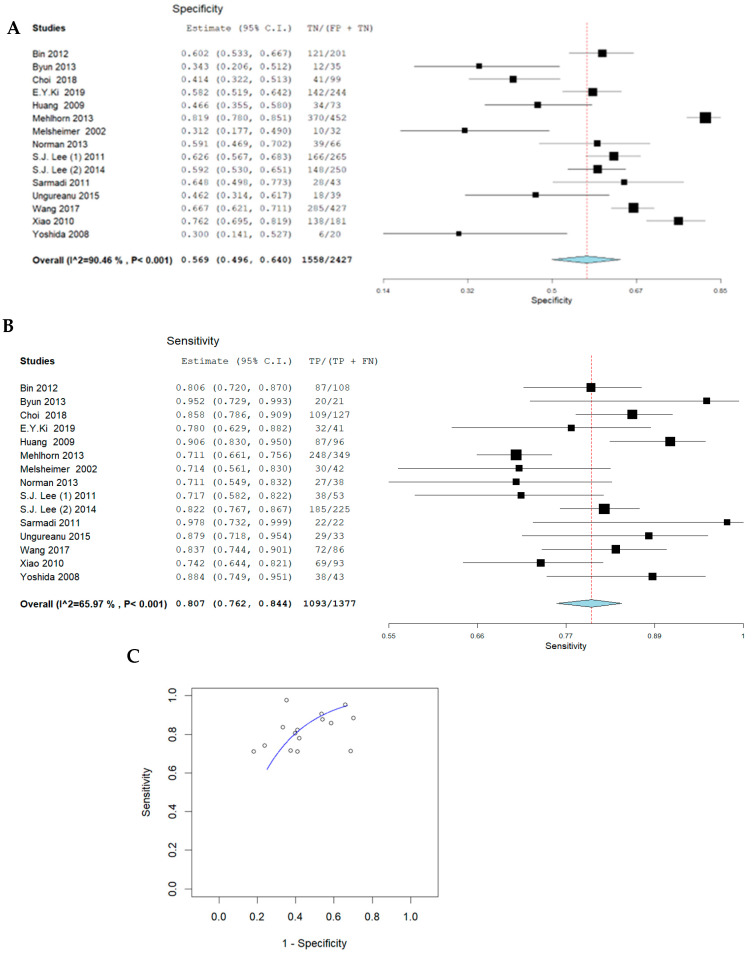
Forest plot of studies (n = 15) examining the sensitivity and specificity of the HPV L1-negative expression for ≥CIN2. (**A**) Specificity; (**B**) sensitivity; and (**C**) summary ROC analysis [9,12,13,17,18,19,20,21,22,23,24,25,26,27,28].

In the analysis, we calculated the deviation index of sensitivity for each study relative to the overall average sensitivity. The resulting funnel plot illustrated a symmetrical distribution, suggesting that smaller studies did not disproportionately report more favorable results compared to larger studies. This symmetry in the funnel plot provided no evidence of publication bias, indicating that the findings were not influenced by the selective reporting of studies (Scheme 1).

**Chart 1:**
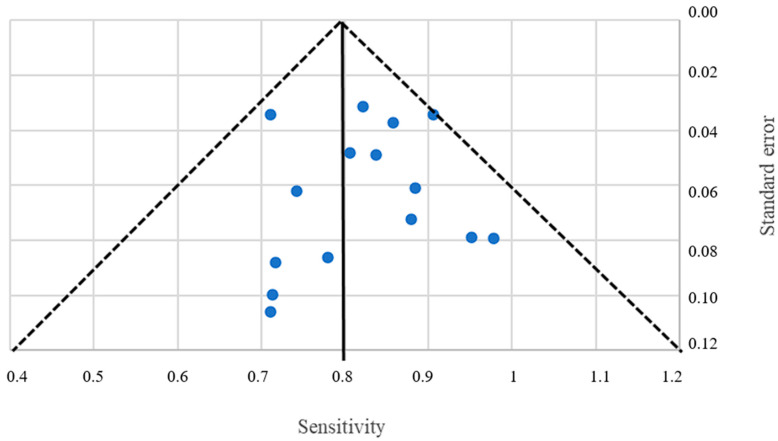
The symmetrical distribution of publication bias. Y = standard error; X = sensitivity.

We assessed publication bias using a funnel plot (Chart 1) and Egger’s test (*p* = 0.12). The funnel plot showed approximate symmetry, and Egger’s test did not reveal statistically significant asymmetry, suggesting a low likelihood of substantial publication bias in our analysis.

### 3.2. Subgroup Analyses

**Scheme 2:**
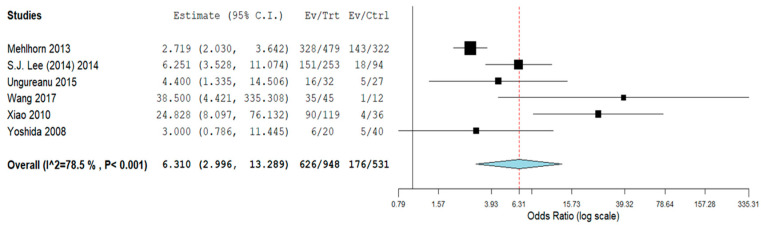
Forest plot of OR L1-positive cases among the LSIL and HSIL cytology groups. The distribution of study weight: Mehlhorn (24.422%) [18], Ungureanu (15.370%) [21], Xiao (16.118%) [23], S.J. Lee (22.074%) [25], Yoshida (13.942%) [27] and Wang (8.073%) [28].

When we categorized the L1-positive cases within each cytological subgroup and conducted a comparative analysis, the combined results from the odds ratio model using the random effects method showed that the detection of HPV L1-positive expression was significantly more frequent in the LSIL group than in the HSIL group (six studies; OR = 6.310, 95% CI: 2.996–13.289, *p* < 0.001). The results are illustrated in Scheme 2. A high level of OR heterogeneity was observed among the included studies (I^2^ = 78.5%, *p* < 0.001), which was statistically significant [18,21,23,25,27,28]. The Mehlhorn study carried the most weight among the six studies in this meta-analysis [18]. The OR from Lee et al. (2013) closely matched the values identified in the meta-analysis [13]. It has been shown that the distinction between LSIL and HSIL is unreliable, as the confidence interval includes values around 1 [27]. Nonetheless, this investigation was of the least significance (13.942%).

**Figure 3:**
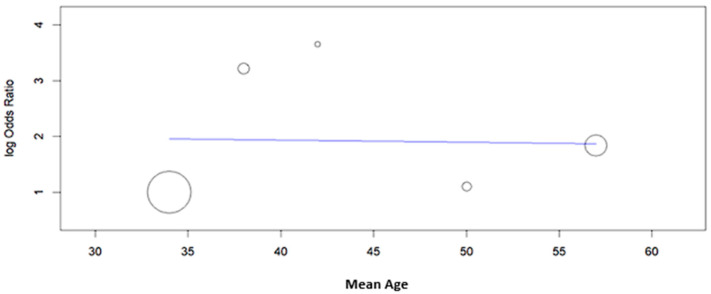
Meta-Regression of mean age (LSIL and HSIL cytology group). Meta-regression of included trials to explore the association between the Mean Age (X) and OR (Y) of L1-positive HPV cases among the LSIL and HSIL cytology group.

A meta-regression was conducted to investigate the relationship between differences in the mean age of women and the OR of HPV L1-positive cases among LSIL and HSIL cytology groups (Figure 3). The regression analysis showed that there was no statistically significant relationship (the coefficient of regression was −0.004 [95% CI −0.094–0.086], *p* = 0.933).

**Scheme 3:**
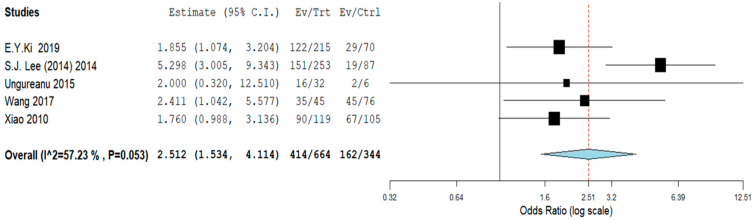
Forest plot of OR L1-positive cases among the LSIL and ASC-US cytology groups. The distribution of study weight: Ungureanu (6.074%) [18], E.Y. Ki (25.797%) [22], S.J. Lee (25.188%) [25], Xiao (24.884%) [23] and Wang (18.057%) [28].

The combined results of the OR model using the random effect method showed that detection of HPV L1-positive expression was statistically significantly more frequent in the LSIL group than in ASC-US (five trials; OR 2.512; 95% CI 1.534 to 4.114; *p* < 0.001) (Scheme 3). (I^2^ = 57.23%, *p* = 0.053) [18,19,20,21,22,23]. Furthermore, most of the studies from the South Asian region have contributed the most significantly. In two trials, it has been demonstrated that this distinction between LSIL and ASC-US is unreliable, as the confidence interval encompasses values of 1 [21,23]. At the same time, the study weight of Xiao’s research was significant [23].

**Figure 4:**
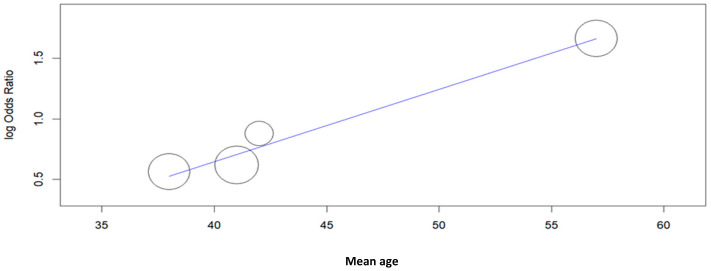
Meta-regression of mean age (LSIL and ASC-US cytology group). Meta-regression of included trials to explore the association between Mean age (Y) and OR (X) of L1-positive HPV cases among the LSIL and ASC-US cytology group.

The meta-regression analysis showed that the mean age of patients has a statistically significant impact on the positive HPV L1 protein expression ratio in the LSIL and ASC-US cytology groups (Figure 4). The regression coefficient was 0.06 (95% CI: 0.021 to 0.099, *p* = 0.003).

This observed dependence can be described by the following equation:

YOR = −1.749 + 0.06 × X

X—mean age, years; YOR—odds ratio.

With each 1-year increase in the mean age of study participants, the odds ratio of detecting the HPV L1 protein in the LSIL cytology relative to the ASC-US cytology is expected to increase by 0.06.

**Scheme 4:**
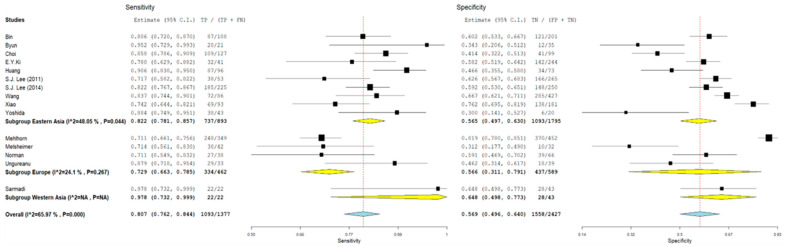
Forest plot of the sensitivity and specificity of L1-negative expression for CIN2+ among the regions [9,12,13,17,18,19,20,21,22,23,24,25,26,27,28].

The data of the meta-analysis on the sensitivity and specificity of immunocytochemical detection of the HPV L1 protein in various research regions are presented in Scheme 4. When analyzing the generalized data by region, the sensitivity of HPV L1-negative expression for predicting CIN2+ ranged from 72.9% in Europe to 97.8% in Western Asia (95%CI: 73.2–99.9%, *p* = NA) in a single study [6,12,18]. Most studies included in the analysis were from Eastern Asia. For this region, the sensitivity was 82% (95CI: 0.77–086, *p* < 0.01), with moderate statistical heterogeneity (I^2^ =48.05%, *p* = 0.044). The specificity was 56.5% (95% CI: 0.497 to 0.630, *p* = 0.061) in Eastern Asia. High statistical heterogeneity of the specificity index was observed in this region, which was statistically significant in the Eastern Asia region (*p* < 0.001, I^2^ = 85.52%). In a meta-analysis of subgroups from the European region, the sensitivity and specificity of L1-negative HPV for CIN2 + were 72.9% (95% CI: 0.663 to 0.785; *p* < 0,001) and 56.6%, respectively (95% CI: 0.311 to 0. 791; *p* = 0.623). In European countries, there was no significant statistical heterogeneity in sensitivity, but there was significant heterogeneity in specificity (*p* = 0.267; I^2^ = 24.1), which was high and statistically significant.

## 4. Discussion

Cervical cytology is a cornerstone of cervical cancer screening and has led to a significant reduction in mortality rates. Although cytology remains an essential tool, its sensitivity for the detection of CIN2+ is limited (62.5% for conventional cytology and 72.9% for liquid-based cytology) [6]. Our results suggest that L1 detection may reduce the risk of missing precancerous lesions compared to cytology alone. However, identifying hrHPV infections at risk of progression is a major challenge, as many hrHPV infections do not lead to cytologic or histologic changes and may often resolve spontaneously [29,30].

This meta-analysis evaluated the diagnostic and prognostic utility of HPV L1 ICC in cervical smears to guide treatment decisions in women of reproductive age. We analyzed fifteen studies (2002–2019), which showed a pooled sensitivity of 80.7% (95% CI: 76.2–84.4%, I^2^ = 65.97%, *p* < 0.001) for predicting CIN2+ in L1-negative cases. Specificity was limited at 56.9% (95% CI 49.6–64%) with significant heterogeneity (I^2^ = 90.46%, *p* < 0.001). Several factors may be responsible for this low specificity. L1 expression may be reduced or absent not only in CIN2+, but also in reactive changes or transient HPV infections that resolve spontaneously. These conditions may be misidentified as CIN2+ on the basis of L1 ICC. Subjectivity in the interpretation of ICC results, as well as differences in the thresholds used to define L1-positive and L1-negative results, may contribute to false-positive results. Nonspecific staining or other technical artifacts can simulate reduced L1 expression and lead to a false-positive result. The variability of methods and reagents can also lead to differences in staining intensity and consequently to a different interpretation of the results. The low specificity has significant implications for clinical practice. The use of L1 ICC as a stand-alone screening test would lead to a large number of false-positive results, resulting in unnecessary colposcopies and biopsies, increased patient anxiety, and unwarranted healthcare costs. Therefore, L1 ICC cannot be considered a replacement for existing screening methods but should be used as an adjunct to other methods, such as cytology and HPV testing, to improve diagnostic accuracy and avoid unnecessary invasive procedures. Although the test shows promise in identifying hrHPV-positive women with squamous intraepithelial lesions (SIL), the high heterogeneity must be carefully considered.

The clinical utility of L1 assessment becomes particularly evident when examining its expression across the CIN2+ continuum—a category encompassing CIN2 (moderate dysplasia), CIN3 (severe dysplasia/carcinoma in situ), and CIS. Our analysis reveals a striking histological gradient: while CIN1 lesions maintain L1 positivity in 68–72% of cases, this drops to 23–41% in CIN2 and plummets to merely 3.1–7.1% in CIN3/CIS. This progression mirrors the biological transition from productive HPV infection to transformative neoplasia, where viral integration typically silences L1 expression.

The gradual erosion of L1 expression across the CIN2+ spectrum reflects fundamental virological transitions. During productive infection (characteristic of CIN1), an intact L1 protein facilitates virion assembly, triggering immune recognition through TLR4/NF-κB pathways. In contrast, HSIL progression involves HPV DNA integration and E6/E7 overexpression, with L1 loss serving as a molecular marker of this transition. Notably, the near-complete L1 absence in CIN3/CIS (96.9% negative in our pooled analysis) versus partial retention in CIN2 suggests this biomarker could help demarcate progression thresholds within the CIN2+ category.

Due to the substantial heterogeneity, a random effects model was used for all pooled analyses. The heterogeneity observed in both sensitivity (I^2^ = 65.97%, *p* < 0.001) and specificity (I^2^ = 90.46%, *p* < 0.001) complicates interpretation and limits generalizability. Potential sources of heterogeneity include variations in study design, sample characteristics, and cytologic/histologic methods. Methodological differences in ICC (assessment of L1 expression, antibodies, and protocols) are probably the main causes. Patient population characteristics (age, ethnicity, location, HPV type prevalence, and screening history) and lesion severity/concomitant infections may also contribute.

Across studies, L1 expression was highest in LSIL (CIN1), which may improve diagnostic accuracy in ambiguous LSIL cases. The variability in sensitivity (71–97%) is likely due to differences in study validity, sample size, participant demographics, and geographic factors. Several studies (Ki, 2019; Lee, 2013; Bin, 2013; Rauber, 2008; Melsheimer, 2003) indicated decreasing L1 protein expression with increasing SIL severity, suggesting potential viral DNA integration [12,20,22,25,29]. A meta-regression revealed no statistically significant relationship between mean age and the probability of HPV L1 positivity when comparing LSIL and HSIL cytology group (regression coefficient: −0.004, [95% CI −0.094–0.086], *p* = 0.933). While this result suggests that age per se has no differential effect on L1 expression associated with LSIL and HSIL, it should be noted that the analysis did not take into account other potentially confounding variables such as HPV viral load, the presence of certain high-risk HPV genotypes, or host immune status [31,32,33].

Meta-regression revealed that the mean age of patients had a statistically significant effect on the ratio of HPV L1 protein expression in LSIL compared to the ASC-US cytology (regression coefficient: 0.06; 95% CI: 0.021 to 0.099, *p* = 0.003). This means that for each year that the mean age increases, the odds of HPV L1 protein detection in the LSIL cytology increase by a factor of 0.06 compared to the ASC-US cytology. This observation could be due to age-related changes in the host immune response or changes in the prevalence of certain HPV genotypes [34,35]. Further studies are needed to determine whether treatment of L1-positive and L1-negative cases in the context of age may improve clinical outcomes.

In addition, the ‘gold standard’ for intraepithelial lesion diagnostics, colposcopy-navigated biopsy with subsequent histopathologic assessment, has its own limitations. Biopsy results can be affected by sampling errors, with small or superficial lesions potentially being missed. In addition, interobserver variability in histopathologic interpretation may contribute to diagnostic discrepancies, particularly in distinguishing between CIN2 and CIN3. The reliance on HSIL+ cytologic diagnosis in a subset of studies (S. J. Lee, 2011; P. Melsheimer, 2002; S. Sarmadi, 2011) when histologic data were unavailable introduces a further degree of uncertainty [9,12,13]. Although HSIL+ cytology has strong predictive power for underlying CIN2+ or higher, there is still a risk of over- or underdiagnosis.

Subgroup analyses by region, age, and study quality were performed. Subgroup analysis by region showed that positive expression of HPV L1 was significantly higher in LSIL than in ASC-US (OR 2.512; 95% CI 1.534 to 4.114; *p* < 0.001), supporting the idea that not all ASC-US cases are consistent with CIN1. There was significant heterogeneity within the regional subgroup (I^2^ = 57.23%, *p* = 0.053). Meta-regression showed that the mean age of patients significantly influenced the relative detection of L1 expression in LSIL compared to ASC-US, with the proportion of L1 positivity being relatively higher in LSIL compared to ASC-US with increasing age.

These results suggest that cytology alone is not sufficient to accurately predict progression to CIN. While L1 expression decreases slightly with age overall (consistent with the trend towards spontaneous HPV resolution), the difference in L1 positivity between LSIL and ASC-US becomes more pronounced in older women. This suggests that in younger women, L1 expression in ASC-US is relatively closer to that of LSIL, which may lead to overtreatment if L1 status is not taken into account [36]. Conversely, older women with LSIL have significantly higher L1 expression than older women with ASC-US, meaning that a more conservative treatment approach (e.g., close monitoring) may be appropriate for this older LSIL group, as their lesions are more likely to regress. The key point is that the differential expression of L1 between these two lesion types and its change with age offer potentially valuable prognostic information beyond cytology alone.

Our regional analysis highlighted the significant contribution of South Asian studies. Interestingly, some of these studies (Xiao, 2010; Ungureanu, 2015) reported that L1 positivity alone was unreliable in distinguishing between LSIL and ASC-US, suggesting that these categories represent a spectrum of cervical changes rather than distinct entities in this region [21,23]. These findings, in conjunction with the observed greater frequency of L1 expression in LSIL compared to HSIL, emphasize the prognostic value of L1-based triaging. Consistent with this hypothesis, other studies (Mehlhorn, 2013) have shown that L1-negative LSIL cases have a higher risk of progression than L1-positive cases; therefore, the absence of L1, as we found in this analysis, should warrant additional screening [18].

These findings suggest that L1-positive cases should be closely monitored by cytology and reflex testing, while L1-negative cases should be further investigated by colposcopy and biopsy. The heterogeneity observed in some studies underscores the recognized challenges in consistent interpretation of cervical lesions and may be due to differences in criteria and practice among pathologists and clinicians. Importantly, age also had no significant effect on the association between L1 and HSIL or LSIL, although we did not explicitly examine or control for other patient-specific variables, such as HPV viral load or the presence of high-risk HPV strains.

Furthermore, our analysis highlights the potential for combined marker approaches to improve the prediction of HSIL+ lesions. Studies using L1 in conjunction with other markers, particularly E2/E6, have shown higher diagnostic accuracy, suggesting that a more holistic approach to biomarker assessment may be warranted [19].

Finally, the observed regional variability in L1 expression should also be carefully considered. Sensitivity for negative L1 expression ranged from 72.9% in Europe to 97.8% in West Asia, with heterogeneity in specificity observed across the European studies. This variability highlights the potential influence of healthcare access and population characteristics on L1 performance and underscores the need for validation studies in different geographic settings to determine the robustness and generalizability of using L1 expression as a prognostic tool. Further studies should investigate which characteristics in these populations influence L1 expression. Strengths of future studies could include stratification and analysis of co-infection with viral variants and better integration with other risk factors. Overall, these results emphasize the complexity of cervical lesion progression and highlight the potential of L1-based testing for individualized patient treatment.

### 4.1. Translating These Insights into Clinical Practice

The biological behavior of L1 directly informs its diagnostic applications within modern classification systems. Our findings validate the WHO 2020 two-tiered paradigm (LSIL/HSIL) while suggesting L1 staining may refine risk stratification, particularly for CIN2 cases where management decisions remain challenging. Specifically, L1 assessment could help (1) identify CIN3/CIS cases requiring urgent intervention, (2) resolve borderline CIN2/C3 diagnoses, and (3) guide conservative management in young patients. This aligns with the LAST project recommendations while adding molecular granularity to histological assessment.

### 4.2. Limitations of the Review

This meta-analysis has several limitations that affect the robustness and generalizability of the results. First, there was significant heterogeneity between the included studies (I^2^ = 78.5% for odds ratios) due to different study designs, populations, and methodologies. This heterogeneity complicates the interpretation of the pooled estimates and urges caution in the clinical application of these results.

Second, the rare L1-positive CIN3 cases (noted in Yoshida et al. and Huang et al.) may represent either technical detection limits or true biological variants, particularly at CIN2-CIN3 transition zones where histological and molecular features often overlap [26,27].

Also, some studies had small sample sizes, which may lead to less reliable effect estimates and reduce statistical power. The Mehlhorn study with the largest sample size particularly affected the pooled odds ratio [18]. The variability of the diagnostic criteria also makes comparison difficult. Moreover, patient demographics, particularly age and geographic location, differed across studies, potentially introducing biases due to variations in HPV prevalence and healthcare practices.

Finally, while funnel plot analysis suggested minimal publication bias, future research should prioritize standardized methodologies to improve the comparability of findings on the relationship between HPV L1 expression and cervical lesions.

## 5. Conclusions

This meta-analysis shows that HPV L1 ICC has a pooled sensitivity of 80.7% (95% CI: 76.2–84.4%, I^2^ = 65.97%, *p* < 0.001) for the prediction of CIN2+ in L1-negative cases of SIL. While this sensitivity is clinically significant for the identification of high-risk patients, the specificity was limited, and heterogeneity was high. Despite this heterogeneity, detection of HPV L1 can potentially help to differentiate between lesions that require treatment and transient HPV infections. Furthermore, as reduced expression correlates with more severe lesions, HPV L1 may be helpful in predicting SIL progression or regression. Further high-quality studies that take heterogeneity into account are needed before clinical application can be recommended.

## Figures and Tables

**Table 1:** The main characteristics of 15 studies examining the immunohistochemical expression of HPV L1 in different cytological and histological groups.

Study	Type of Study	Mean Age ± SD or (Range), Years	N° of Cases	Cytology (n)	Histology (n)	HPV L1-Positive Cases (n)		Key Findings
						Cytology (n)	Histology (n)	
S. J. Lee, 2011, Korea [9]	PCS	L1+: 41.8 ± 10.1 L1−: 40.9 ± 11.2	318	LSIL (318)	ND	LSIL (181)	ND	Higher HSIL risk in L1-negative; PPV of L1-positive for no progression 91.7%; NPV of L1-negative for HSIL progression 27.7%.
P. Melsheimer, 2002, Germany [12]	PCS	36.5 ± 11.3	74	LSIL (32); HSIL (42)	ND	LSIL (10) HSIL (12)	ND	Reduced HPV L1 expression in HPV 16 DNA-positive HSIL.
S. Sarmadi, 2011, Iran [13]	RCS	38.9 ± 10.7	65	HSIL (22) LSIL (43)	ND	HSIL (22): remission (3) persistence (6) LSIL (28): remission (17) persistent (9) progression (2)	ND	Significant association between HPV L1 positive staining and lack of progression in LSIL. Women under the age of 30 had a significantly higher frequency of positive L1 staining.
S. W. Byun, 2013, Korea [17]	PCS	46 (25–83)	56	LSIL (8) ASC-H (26) LSIL-H (30) HSIL (8)	Chronic cervicitis (12) CIN1 (8) CIN2 (5) CIN3 (23)	LSIL (3) HSIL (1) ASC-H LSIL-H (13)	<CIN2 (12) ≥CIN2 (1)	HPV L1 negativity is associated with CIN2+ in follow-up but not with histology; low specificity and PPV for predicting CIN2+. The HPV L1 protein sensitivity and specificity for predicting follow-up CIN2+ were 95.2%, 34.3%, 46.5%, and 92.3%, respectively.
G. Mehlhorn, 2013, Germany [18]	PCS	33.6 (range 15–83)	908	LSIL (479) HSIL (322)	CIN1 CIN2 CIN3+	471 HPV L1-positive	LSIL (328) HSIL (143) etc.	Significantly higher spontaneous remission in L1-positive LSIL; higher progression risk in L1-negative.
Y.-J. Choi, 2018, Korea [19]	RCS	Normal: 37.8 CIN1: 37.3 CIN2/3: 38.6 SCC: 52.6	226	Normal (21) ASCUS (53) LSIL (66) HSIL (49) SCC (37)	Normal (57) CIN1 (42) CIN 2/3 (69) SCC (48)	ND	Normal (27) CIN1 (14) CIN 2/3 (17) SCC (1)	Significantly lower HPV L1 positivity in CIN2/3 and SCC vs. normal tissue; poor discrimination between CIN1 and CIN2/3 (*p* < 0.001; 95% l CI = 0.071–0.445). The sensitivity and specificity of the combination of the HPV E2/E6 ratio and HPV L1 were 99.2% and 31.3%, respectively.
H. Bin, 2012, China [20]	RCS	37.3 ± 8.9	309	NILM (72) ASCUS (71) LSIL (80) ASC-H (19) HSIL (49) SCC (18)	Normal (33) CIN 1 (168) CIN 2/3 (84) SCC (24)	ND	Normal (9) CIN 1 (112) CIN 2/3 (21) SCC (0)	HPV L1 negativity correlated with higher-grade cervical lesions. (rs = −0.272, *p* < 0.001).
C. Ungureanu, 2015, Romania [21]	PCS	ND	76	NILM (8) ASC-US (6) LSIL (32) HSIL (27) SCC (3)	Normal (12) CIN1 (31) CIN2 (17) CIN3 (13) SCC (3)	NILM (1) ASC-US (2) LSIL (16) HSIL (5) SCC (0)	ND	L1-/p16-pattern frequent in NILM; moderate diagnostic accuracy. The diagnostic test results were Sn = 87.88%, Sp = 46.51%, PPV = 55.8%, and NPV = 83.3%.
E. Y. Ki, 2019, Korea [22]	RCS	40.7 (20–78)	285	ASC-US (70) LSIL (215)	CIN1 or cervicitis (244), CIN2+ (41)	ASC-US (29) LSIL (122)	ND	HPV L1 (−) ASC-US/LSIL and HPV 16/18 had a higher risk of CIN2+ than HPV L1 (+). HPV L1 (−) ASCUS progressed to CIN2+ more frequently than LSIL. HPV L1 (−) was more common with other HPV types.
W. Xiao, 2010, China [23]	PCS	37.99 ± 9.40	274	ASC-US (105) LSIL (119) ASC-H (9) HSIL (36) SCC (6)	Cervicitis (96) CIN 1 (85) CIN 2 (55) CIN 3 (32) SCC (6)	LSIL (90) ASC-US (67) ASC-H (1) HSIL + and SCC (4)	Cervicitis (67) CIN 1 (71) CIN 2 (23) CIN 3 (1) SCC (0)	HPV L1 (+) was more frequent in CIN 1 than in CIN 2 and higher in the ASCUS group. Sensitivity of L1 (−) for ≥CIN2 was 74.19%, specificity was 76.24%, and NPV was 85.18%. Regression at 1 year was more frequent with HPV L1 (+).
I. Norman, 2013, Sweden [24]	RCS	32 (23–57)	104	WNL (ND) LSIL (ND)	Normal (23) CIN1 (43) CIN2 (23) CIN3+ (15)	ND	Normal (13) CIN1 (26) CIN2+ (11)	L1-negative cases are significantly associated with higher CIN grade and progression to CIN2+ (OR 3.2 [95% CI, 1.081–9.417]).
S. J. Lee, 2014, Korea [25]	RCS	56.6	475	ASC-US (87) LSIL (253) HSIL (94) SCC (41)	≤CIN1 ≥CIN2 ≤CIN2 ≥CIN3	ASC-US (19) LSIL (151) HSIL (18) SCC (0)	CIN 1 (148) ≤CIN2 (164) ≥CIN2 (40) ≥CIN3 (24)	Highest HPV L1 positivity in LSIL (*p* < 0.0001); high percentage of L1-negative cases in ASC-US; higher risk of ≥CIN2 progression in L1-negative ASCUS and LSIL
M.-Z. Huang, 2009, China [26]	RCS	35.09 ± 8.9	169	LSIL HSIL SCC	CIN1 (73) CIN2 (41) CIN3 (28) SCC(27)	ND	CIN1 (34) CIN2 (7) CIN3 (2) SCCs (0)	HPV L1 expression decreased with histological grade; combining L1 with p16 improved specificity. (*x*^2^ = 32.86, *p* < 0.001).
T. Yoshida, 2008, Japan [27]	RCS	50 (24–74)	63	LSIL (20) HSIL (40) SCC (3)	CIN2 SCC	LSIL (6) HSIL (5) SCC (0)	ND	L1(−)/p16(+) is highly prevalent in HSIL and SCC; L1(+)/p16(−) is rare.
J.-J. Wang, 2017, China [28]	PCS	42.19 ± 8.06	513	NILM (368) ASCUS (76) LSIL (45) ASC-H (12) HSIL (12)	Normal (312) CIN1 (15) CIN2 (57) CIN3 (28) ICC (1)	NILM (216) ASCUS (45) LSIL (35) ASC-H (2) HSIL (1)	<CIN2 (285) CIN2+ (14)	No correlation between HPV L1 positivity and viral load; higher sensitivity and NPV but lower specificity in the CIN2+ group for HPV L1 detection. (*p* > 0.05).

PCS—prospective cohort study; RCS—retrospective study; WNL—NILM according to TBS; CI—confidence interval; ASC-H—atypical squamous cell cannot exclude HSIL; Sp—specificity; Sn—sensitivity; PPV—positive predictive value; and NPV—negative predictive value; ND—no data.

## Abbreviations

The following abbreviations are used in this manuscript:

ASC-H	Atypical squamous cell cannot exclude HSIL
ASC-US	Atypical squamous cells of undetermined significance
CI	Confidence interval
CIN	Cervical intraepithelial neoplasia
CIN2+	High-grade cervical intraepithelial neoplasia
HPV	Human papillomavirus
HSIL	High-grade squamous intraepithelial lesions
ICC	Immunocytochemistry
LSIL	Low-grade squamous intraepithelial lesions
NILM	Negative for intraepithelial lesion or malignancy
NPV	Negative predictive value
OR	Odds ratio
PCS	Prospective cohort study
PPV	Positive predictive value
RCS	Retrospective cohort studies
RCT	Randomized clinical trials
Sn	Sensitivity
Sp	Specificity
TBS	The Bethesda System
WNL	NILM according to TBS
